# High-Salt Attenuates the Efficacy of Dapagliflozin in Tubular Protection by Impairing Fatty Acid Metabolism in Diabetic Kidney Disease

**DOI:** 10.3389/fphar.2021.741087

**Published:** 2021-12-20

**Authors:** Meina Zou, Yanrong Chen, Zongji Zheng, Shuyue Sheng, Yijie Jia, Xiangyu Wang, Shijing Ren, Yanling Yang, Xiaomin Li, Wenhui Dong, Meiping Guan, Qian Zhang, Yaoming Xue

**Affiliations:** Department of Endocrinology and Metabolism, Nanfang Hospital, Southern Medical University, Guangzhou, China

**Keywords:** diabetic kidney disease, high-salt, renal tubule, fatty acid metabolism, Na+/K+-ATPase, dapagliflozin

## Abstract

High-salt intake leads to kidney damage and even limits the effectiveness of drugs. However, it is unclear whether excessive intake of salt affects renal tubular energy metabolism and the efficacy of dapagliflozin on renal function in diabetic kidney disease (DKD). In this study, we enrolled 350 DKD patients and examined the correlation between sodium level and renal function, and analyzed influencing factors. The results demonstrated that patients with macroalbuminuria have higher 24 h urinary sodium levels. After establishment of type 2 diabetes mellitus model, the animals received a high-salt diet or normal-salt diet. In the presence of high-salt diet, the renal fibrosis was aggravated with fatty acid metabolism dysregulation. Furthermore, Na+/K+-ATPase expression was up-regulated in the renal tubules of diabetic mice, while the fatty acid metabolism was improved by inhibiting Na+/K+-ATPase of renal tubular epithelial cells. Of note, the administration with dapagliflozin improved renal fibrosis and enhanced fatty acid metabolism. But high salt weakened the above-mentioned renal protective effects of dapagliflozin in DKD. Similar results were recapitulated *in vitro* after incubating proximal tubular epithelial cells in high-glucose and high-salt medium. In conclusion, our results indicate that high salt can lead to fatty acid metabolism disorders by increasing Na+/K+-ATPase expression in the renal tubules of DKD. High salt intake diminishes the reno-protective effect of dapagliflozin in DKD.

## Introduction

The World Health Organization recommended to reduce salt intake to less than 5 g/day of salt (Brown et al., 2009). The National Kidney Foundation Kidney Disease Outcomes Quality Initiative (NKF/KDOQI) also supports the recommendation of salt intake restriction (Inker et al., 2014). However, salt consumption is still high (Brown et al., 2009). A high-salt intake is associated with the development of many diseases. The effect of salt on cardiovascular disease has been fully elucidated (Bibbins-Domingo et al., 2010; He et al., 2000; O’Donnell et al., 2014). Based on the 24-h urine sodium (24 h-UNa) excretion, it was estimated that average salt intake of chronic kidney disease (CKD) patients is 9.5 g/day (de Borst and Navis, 2016). Altered dietary salt intake is noted to prevent and treat CKD (Lankhorst et al., 2016; Mcmahon et al., 2021). A high-salt diet (HSD) leads to increased biomarkers of renal tubular damage, not associated with hypertension (Hosohata, 2017; Washino et al., 2018), and such a diet may interfere with drugs’ effectiveness (Ekinci et al., 2009; Slagman et al., 2011; Juncos et al., 2012; Kwakernaak et al., 2014). Although the relationship between kidney damage and salt intake is well known, the underlying mechanism is not fully clear and needs to be clarified, especially in type 2 diabetes mellitus (T2DM) and diabetic kidney disease (DKD). Previous studies have suggested that fatty acid metabolism is a main energy source of proximal tubular epithelial cells (PTEC) (Ekinci et al., 2009; Slagman et al., 2011; Juncos et al., 2012; Kwakernaak et al., 2014). In humans and animal models with renal fibrosis, fatty acid oxidation (FAO) was decreaed (Kang et al., 2015; Chung et al., 2018). Many results suggest that impaired renal glucocorticoid receptor (Srivastava et al., 2021), peroxisome proliferator-activated receptor γ (PPARγ) co-activator 1α (PGC-1α) (Han et al., 2017) and sirtuin 3 (SIRT3) (Li et al., 2020) signaling pathways aggravate renal injury by mitigating FAO in kidneys. Furthermore, suppression of N-acetyl-seryl-aspartyl-lysyl-proline (AcSDKP) disrupts kidney cell metabolism and leads to severe fibrosis in the diabetic kidney (Srivastava et al., 2020). However, Na+/K+-adenosine triphosphatase (Na+/K+-ATPase), which maintains cell homeostasis by mainly transporting Na + ions, plays a key role in renal tubules (Katz and Epstein, 1967; Katz and Lindheimer, 1973). In the kidney, the consumption of ATP and oxygen mainly depends on the energy required for the reabsorption of Na+ by the renal tubules (Körner et al., 1994; Layton and Vallon, 2018). Therefore, in this study, we explored the regulatory role of abnormal tubular energy metabolism in the renal fibrosis exacerbated by high-salt in DKD.

Several studies have reported that a variety of hypoglycemic drugs such as sodium-glucose cotransporter protein 2 (SGLT-2) inhibitors (Perkovic et al., 2019), dipeptidyl peptidase 4 (DPP-4) inhibitors (Rosenstock et al., 2019; Perkovic et al., 2020), glucagon-like peptide 1 (GLP-1) receptor agonists (Gerstein et al., 2019) exert beneficial effects on renal function. Besides, angiotensin-converting enzyme inhibitor and/or angiotensin II receptor blocker (ACEi/ARB) is also considered renal-protective (Gayat et al., 2018). However, no study has evaluated the associations between salt intake and the efficacy of DKD treated with SGLT2 inhibitors (such as dapagliflozin). Studies have indicated that empagliflozin attenuates albuminuric in db/db diabetic mice (Klimontov et al., 2020) while inhibition of SGLT2 in the kidney proximal tubules may exert renal protective effect through energy metabolism (Layton and Vallon, 2018; Bessho et al., 2019). Thus, further exploration of the effect of high salt on the dapagliflozin efficacy provides a theoretical basis for the salt diet management during the treatment of DKD.

## Methods

### Clinical Study

A retrospective study was conducted at the Department of Endocrinology and Metabolism, Nanfang Hospital of Southern Medical University, involving 350 DKD patients ≥ 18 years of age with an eGFR ≥ 15 ml/min/1.73 m^2^ and urine albumin-to-creatinine ratio (ACR > 3.4 mg/mmol). Exclusion criteria were as follows: patients suffered from type 1 diabetes mellitus (T1DM), acute complications of diabetes, advanced renal failure, heart disease, urinary infection, cerebrovascular accident, kidney transplantation, and other confirmed renal disease (e.g., polycystic kidney, solitary kidney), and pregnant and lactating women. This study was approved by the Medical Ethics Committee of Nanfang Hospital of Southern Medical University, Guangzhou, China (Certificate Number: NFEC-2021-144).

### Animal Research

Forty-two 7-week-old C57BL/6J male mice were obtained from the Guangdong Medical Laboratory Animal Center (Guangdong, China). Thirty mice (DM group) were administrated a single injection of streptozotocin (STZ, 120 mg/kg, i.p., pH = 4.5, Sigma-Aldrich, United States) dissolved in citrate buffer after a high-fat diet (HFD, D12492, HFK Bioscience, Beijing, China) for 4 weeks (Jia et al., 2019; Yang et al., 2020). A sustained blood glucose level, measured once a week, of > 16.7 mM indicated hyperglycemia. The other mice (*n* = 12) received injection of equal volume of sodium citrate.

Two weeks after STZ injection, DM group mice were randomly divided into two groups: 1) DM + HNa group mice (*n* = 12) were given HFD and HSD (60% fat and 4.0% salt, Guangdong Medical Animal Experimental Center, Guangdong, China); 2) DM group mice (*n* = 18) were fed with HFD and normal salt diet (NSD, 0.5% salt). Control mice were also randomly divided into two groups: 1) Control + HNa group mice (*n* = 6) were given HSD (Guangdong Medical Animal Experimental Center, Guangdong, China); 2) Control group mice (*n* = 6) were fed with NSD.

After 8 weeks HSD, mice were divided into the following groups: DM + Saline group (*n* = 6), DM + Dapa group (*n* = 6), DM + HNa + Dapa group (*n* = 6). The above mice were administrated with saline or dapagliflozin (1 mg/kg/day, MedChemExpress, China) by gavage for 12 weeks and continued their previous diet. All *in vivo* studies were performed from 2 to 4 pm, and approved by the Animal Ethics Committee of Southern Medical University, Guangzhou, China (Certificate Number: SYXK 2016-0167).

### Intraperitoneal Glucose Tolerance Test

After 4 weeks HFD and intraperitoneal injection of STZ, IPGTT was conducted to evaluate the glucose responsiveness of mice. In short, mice (*n* = 6 per group) were fasted for 16 h with free access to drink water. Mice were injected intraperitoneally with glucose (2 mg/g body weight). Blood glucose levels were measured at 0, 15, 30, 60, 90 and 120 min.

### Intraperitoneal Insulin Tolerance Test

Before the mice were sacrificed, IPITT was conducted to evaluate the insulin tolerance after mice were fasted for 6 h with free access to drink water. Mice (*n* = 6 per group) were injected intraperitoneally with insulin (0.75 U/kg body weight). Blood glucose levels were measured at 0, 15, 30, 60, 90 and 120 min.

### Urinary Albumin, Creatinine, Na and Serum Na

For collecting urine, all experimental mice were housed individually in metabolic cages for 24 h with drinking water, without food. The mice were allowed to recover for 24 h before performing other operations. The urine output and water consumption were also measured. The urinary albumin (ml025061-j2; MIBio, Shanghai, China) and creatinine (ml026283-j2; MIBio, Shanghai, China) were detected by ELISA according to the manufacturer’s instructions. After fasting for 12 h, serum was collected. The Na levels in the urine and serum were measured by sodium assay kit (Nanjing Jiancheng Bioengineering Institute, Jiangsu, China) using a SpectraMax M5 Multifunctional Microplate Reader (Molecular Devices, United States).

### Cell Culture and Transfection

Human renal tubular epithelial cell line HK-2 (China Center For Type Culture Collection) was cultured in 1.0 g/L Dulbecco’s modified Eagle medium (DMEM) containing 10% fetal bovine serum (FBS). After starvation with 2% FBS for 12 h, cells were cultured in 4.5 g/L DMEM medium, whether containing 15 mM NaCl (provided in the form of NaCl) or not, to simulate high glucose and salt conditions *in vivo*. Simultaneously, 15 mM sodium gluconate (Aladdin, Shanghai, China) or 25, 30 and 55 mM mannitol (Fuchen, Tianjing, China) were used to intervene cells to eliminate the influence of osmotic pressure and chloride ions. After culture, cells were treated with 5 nM Digoxin (APExBIO, United States), 40 μM Etomoxir (MedChemExpress, China) or 5 μM dapagliflozin and collected for corresponding examination.

The small interfering RNA targeting SLC5A2 (siSLC5A2) and the appropriate negative control (siNC) (Ribobio, Guangzhou, China) were transfected into HK-2 cells at a final concentration of 50 nM for 72 h. The sequences of si-SLC5A2 are shown in Supplementary Figure S1C. All transfections were conducted with Lipofectamine® 3000 (Invitrogen, Carlsbad, CA, United States) as instructed by the manufacturer’s protocols.

### Kidney Histopathology and Immunohistochemistry

The renal cortex was fixed with 10% neutral formalin for 20–24 h, dehydrated and embedded in paraffin (Leica, German). The paraffin was cut into sections and tissue sections were subjected to Periodic Acid-Schiff (PAS) and Masson’s trichrome (Maiwei, Xiamen, China) staining as previously described (Wang et al., 2015). For immunohistochemical analysis, the sections were incubated with primary antibodies at 4°C overnight, including anti-FN (1:300; F3648; Sigma, United States), anti-CPT1A (1:800; 15184-1-AP; proteintech, United States), anti-ACOX1 (1:900; 10957-1-AP; proteintech), anti-FABP4 (1:50; 12802-1-AP; proteintech), anti-FASN (1:350; 10624-2-AP; proteintech), anti-SGLT2 (1:2,000; A03748-1; BOSTER, China), anti-ATP1A1 (1:200; 14418-1-AP; proteintech) and anti-ATP1B1 (1:50; 15192-1-AP; proteintech). They then were probed with secondary antibodies (pv-6001; ZSGB-BIO). The sections were photographed under a BX51 upright microscope (×200 or ×400) with four random fields and analyzed by the ImageJ software.

### Western Blot

Total proteins were extracted using a RIPA lysis buffer (KeyGen BioTECH, Nanjing, China) and the protein concentration was measured by bicinchoninic acid (BCA) assay kit (TAKARA, Dalian, China). Proteins (20 μg) were separated using 10% sodium dodecyl sulphate–polyacrylamide gel electrophoresis (SDS-PAGE) and then transferred to polyvinylidene difluoride (PVDF) membranes (Millipore, Billerica, MA). The membranes were blocked with 10% skimmed milk powder (Sigma, United States) for 1 h and incubated with following primary antibodies overnight at 4°C: anti-FN (1:2000, 66042-1-Ig, Proteintech Group, Inc. Rosement, United States), anti-COL-1 (1:1,000, A16891, ABclonal, Wuhan, China), anti-αSMA (1:500, A7248, ABclonal), anti-TGF-β1 (1:500, A18692, Santa Cruz, California), anti-ATP1A1 (1:5,000, 14418-1-AP, Proteintech), anti-ATP1B1 (1:1,000, 15192-1-AP, Proteintech), anti-CPT1A (1:1,000, 15184-1-AP, Proteintech), anti-ACC (1:10,000, 67373-1-Ig, Proteintech), anti-FASN (1:500, 10624-2-AP, Proteintech), anti-PGC-1α (1:1,000, A12348, ABclonal), anti-SGLT2 (1:600, 24654-1-AP, Proteintech), and anti-β-actin (1:2,000, 66009-1-Ig, Proteintech). The membranes then were probed with goat anti-mouse and anti-rabbit secondary antibodies for 2 h at room temperature. The bands were developed with enhanced chemiluminescent liquid (Millipore) and photographed. The images were analyzed with ImageJ software. We also validated SGLT2 rabbit polyclonal antibody after silencing SLC5A2 in HK-2 cells in Supplementary Figures S1A–C.

### Real-Time Quantitative Polymerase Chain Reaction

Total RNA was extracted using Trizol (TAKARA) and RNA quality was assessed by measuring the OD260/OD280 ratio using a NanoDrop micro spectrophotometer (Thermo Fisher). 1 ug RNA was reversely transcribed into cDNA with a PrimeScriptTMRT reagent Kit (TAKARA) to reverse transcribe RNA to cDNA. RT-qPCR was performed on Roche 480 PCR machine with 10 ul system of RT-qPCR reaction solution, namely 5 ul SYBR® Select Master Mix (2X) (TAKARA), 0.2 ul Forward Primers, 0.2 ul Reverse Primers, 1 ul cDNA and 3.6 ul dH2O. Then operate according to the following RT-qPCR reaction conditions: pre-denaturation at 95°C for 30 s, cycle at 95°C for 5 s and 60°C for 35 s for 50 times. The above experiment was repeated three times. The gene expressions of mouse and human were respectively normalized to β-actin and 18S mRNA and expressed as fold change. All primer sequences are presented in Supplementary Table S1.

### Statistical Analysis

#### Clinical Study

The measurement data of the normal distribution that meets the requirements were expressed as mean ± standard deviation (SD), while those that do not meet the requirements were expressed as median ± interquartile range (IQR). The data of counting were expressed by sample number and composition ratio. The pairwise comparison of normally distributed data uses two independent sample t-tests. The Mann-Whitney test was used to analyze the non-normally distributed continuous data. The data of two groups were compared using the χ^2^ test. The non-parametric test of multiple groups of samples was used by the Kruskal-Wallis test. Binary logistic regression analysis was performed to examine the interaction between following variables and urinary albumin excretion: age, sex, diabetes duration, body mass index (BMI), blood pressure, 24 h urinary sodium, 24 h urinary potassium, serum creatinine, serum uric acid, etc. *p* < 0.05 was considered statistically significant.

### Animal and Cell Research

Data were expressed as mean ± SEM. The pairwise comparison of normally distributed data used two independent sample t-tests, and the difference among multiple group was analyzed by one-way analysis of variance (One-Way ANOVA). Perform Levene’s homogeneity of variance test and analysis of variance first. If the variances are uniform (*p* > 0.05) and there is a significant difference in the means between groups (*p* < 0.05), the LSD method is further used for multiple comparisons; if the variances are not uniform (*p* < 0.05), the approximate F test Welch method is selected for correction; if there is a significant difference in the means between groups (*p* < 0.05), Dunnett’s T3 method is used for multiple comparisons. *p* < 0.05 was considered statistically significant.

## Results

### 24 h-UNa is Higher in DKD Patients With Macroalbuminuria

**FIGURE 1:**
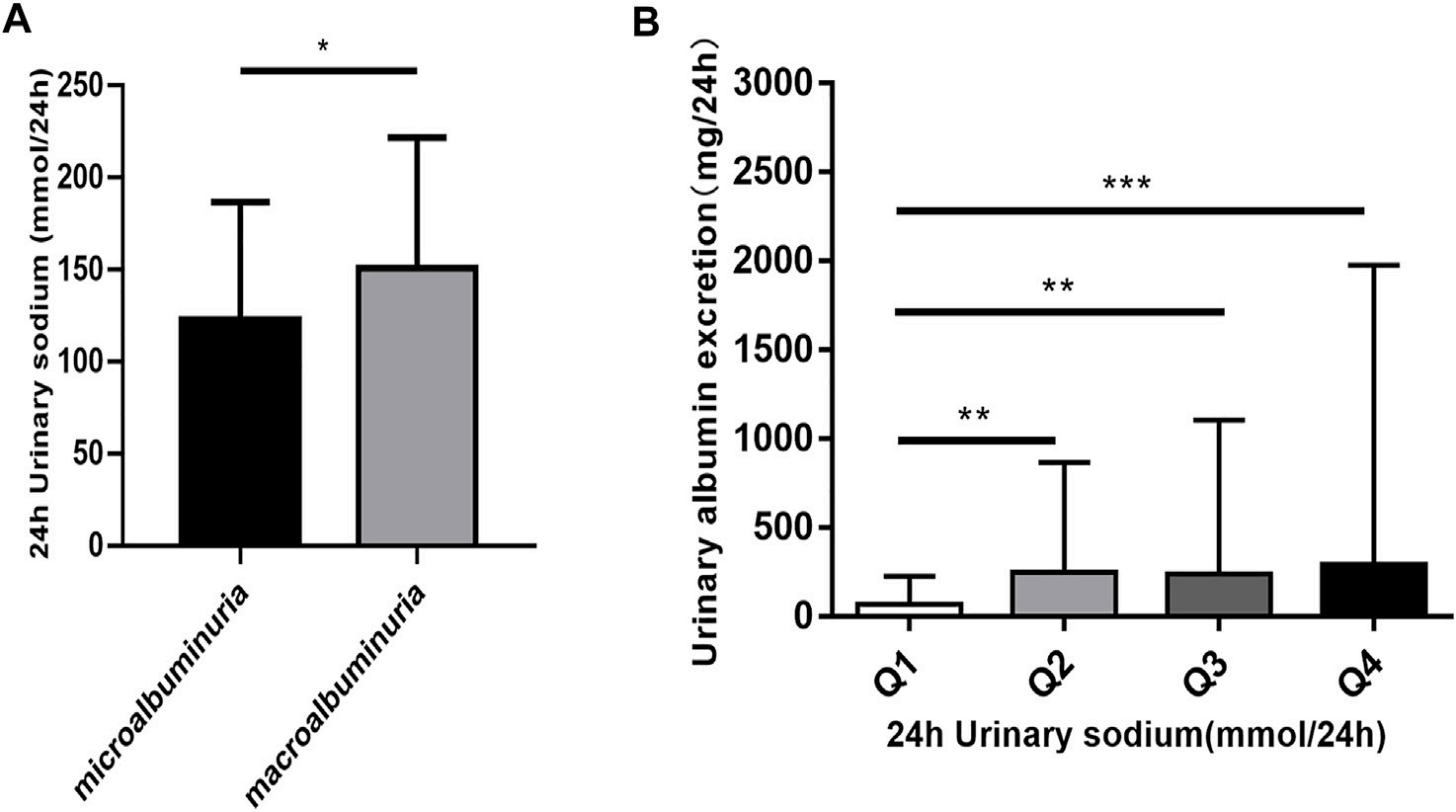
The relationship of 24 h urinary sodium excretion and 24 h urinary albumin excretion. **(A)** Patients were classified according to the urinary albumin excretion. The data are mean ± SD. *p < 0.05 vs. microalbuminuria group. **(B)** The patients were divided into four groups according to 24 h urinary sodium excretion. Quartile 1 (Q1) refers to the lowest sodium excretion quartile, and quartile 4 (Q4) indicates the highest sodium excretion quartile. The data are mean ± IQR. **p = 0.001 and ***p = 0.000 vs. Q1 group.

To assess the potential relationship between 24 h-UNa excretion and renal function in DKD, we collected clinical data from 350 DKD patients, and evaluated the association between 24 h-UNa levels and renal function. Patients were grouped according to the degrees of albuminuria. Compared to the 24 h-UNa excretion of microalbuminuria group, patients of macroalbuminuria group had a higher urinary sodium excretion (Table 1, Figure 1A). Men were more likely to develop macroalbuminuria rather than microalbuminuria. Compared to the patients with microalbuminuria, patients with macroalbuminuria had a longer diabetic duration and higher systolic blood pressure, as we ll as a lower level of glycated hemoglobin (HbA1c), fasting blood glucose and low-density lipoprotein (LDL) with no significant difference on diastolic blood pressure between two groups. Besides, the levels of indexes of renal function including eGFR, serum creatinine and ACR were consistently correlated to the condition of albuminuria (Table 1). Binary logistic regression analysis indicated that LDL-cholesterol, duration of diabetes, systolic blood pressure, serum uric acid and 24 h-UNa excretion were all significantly associated with the degree of albuminuria. Interestingly, our data suggested that age was not the risk factor for DKD (Table 2). We equally divided the patients into four groups according to the excretion of 24-h urinary sodium, and we found that the excretion rate of urinary microalbumin was higher in the other three groups than in the lowest urinary sodium excretion group, but there was no significant increase in urinary protein level with the increase of urinary sodium level (Figure 1B). These data suggest that the excretion of urinary protein may be correlated with the excretion level of urinary sodium, but its correlation needs further validation.

**TABLE 1:** Characteristics of 350 patients with DKD according to 24 h urinary albumin excretion.

Variable	Urinary albumin excretion (mg/24 h)			
	*N* = 350	<300	≥300	*P*
		*N* = 202	*N* = 148	
Age (years)	59 (10)	59 (10)	58 (9)	0.488
Men (%)	225 (64)	114 (56)	111 (75)	0.000*
BMI (kg/m^2^)	24.5 (3.7)	24.4 (3.8)	24.7 (3.7)	0.483
Diabetes duration (years)	11 (7)	10 (7)	13 (7)	0.003*
SBP (mmHg)	141 (19)	139 (18)	145 (20)	0.001*
DBP (mmHg)	81 (11)	80 (11)	82 (11)	0.250
HbA1C (%)	9.1 (2.4)	9.5 (2.4)	8.6 (2.2)	0.001*
FBG (mmol/L)	8.2 (5.1)	8.7 (5.6)	7.5 (4.3)	0.031*
Total cholesterol (mg/dl)	4.9 (1.5)	4.8 (1.6)	5.0 (1.4)	0.140
LDL-cholesterol (mg/dl)	3.1 (1.0)	3.0 (1.0)	3.2 (1.0)	0.013*
HDL-cholesterol (mg/dl)	1.1 (0.4)	1.1 (0.5)	1.0 (0.3)	0.201
Triglyceride (mg/dl)	2.4 (2.3)	2.4 (2.5)	2.4 (1.9)	0.905
Serum creatinine (mg/dl)	102.6 (60.7)	78.1 (32.2)	136.2 (73.3)	0.000*
eGFR (ml/min/1.73 m^2^)	76.3 (30.4)	87.6 (24.7)	60.7 (30.6)	0.000*
Serum uric acid (mg/dl)	403.6 (116.2)	377.0 (112.7)	440.1 (111.2)	0.000*
CRP (mg/L)	1.6 (0.7–3.8)	1.5 (0.7–3.6)	1.8 (0.7–4.6)	0.588
Urinary albumin excretion (mg/24 h)	196.5 (63.0–925.3)	70.5 (45.0–138.3)	1,176.0 (622.5–3238.5)	0.000*
ACR (mg/g)	20.7 (7.6–90.4)	8.7 (5.4–15.9)	117.2 (52.3–284.9)	0.000*
24 h urinary sodium (mmol/24 h)	134.6 (68.4)	122.8 (63.9)	150.6 (71.2)	0.000*
24 h urinary potassium (mmol/24 h)	37.2 (17.2)	34.5 (17.2)	40.8 (16.6)	0.001*

Values are the percentage of participants, mean (SD), or median (IQR). Statistical significance *p < 0.05.

Note: BMI, body mass index; SBP, systolic blood pressure; DBP, diastolic blood pressure; FBG, fasting blood glucose; LDL, low-density lipoprotein; HDL, high-density lipoprotein; eGFR, estimated glomerular filtration rate; CRP, C-reactive protein; ACR, urinary albumin to creatinine rate; SD, standard deviation; IQR, interquartile range.

**TABLE 2:** Multivariable-adjusted ORs and 95% CIs for patients with albuminuria.

Variable	OR (95% CI)	*P*
Diabetes duration (years)	1.042 (1.003–1.082)	0.035
SBP (mmHg)	1.017 (1.001–1.033)	0.040
Serum creatinine (mg/dl)	1.021 (1.014–1.029)	0.000
LDL-cholesterol (mg/dl)	1.510 (1.115–2.047)	0.008
24 h urinary sodium (mmol/24 h)	1.009 (1.004–1.013)	0.000

The ORs and 95% CIs were calculated by using binary logistic models.

Adjusting for patients’ age, sex, BMI, DBP, HbA1C%, FBG, total cholesterol, HDL-cholesterol, triglyceride, CRP, 24 h urinary potassium.

Note: OR, odds ratio; CI, confidence interval.

### High-Salt Diet Aggravates Renal Fibrosis in Diabetic Mice

**FIGURE 2:**
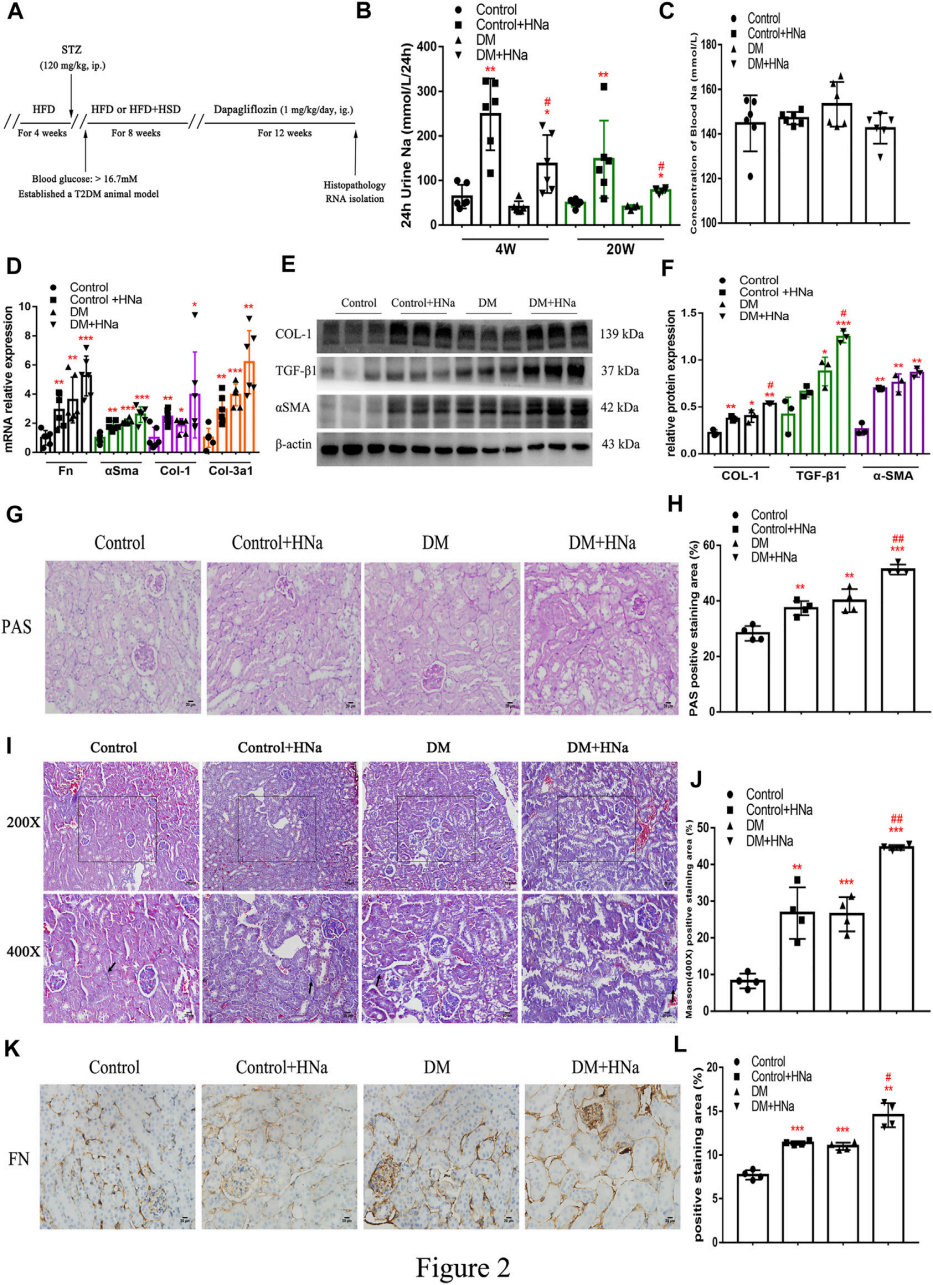
**|** High-salt diet aggravates renal fibrosis in diabetic mice. **(A)** Schematic diagram of the experiment plan. After HFD for 4 weeks, intraperitoneal injection of STZ was performed. One week later, blood glucose was detected and diabetic mice were selected to be fed with high-salt diet for 8 weeks, followed by dapagliflozin by gavage for 12 weeks. **(B)** 24 h urine Na was measured at 4 weeks and 20 weeks after high-salt diet (*n* = 6 per group). **(C)** Blood Na was measured at 20 weeks after high-salt diet (*n* = 6 per group). **(D)** The levels of *Fn,* α*Sma, Col-1 and Col-3a1* were detected by RT-qPCR (*n* = 6 per group). **(E,F)** Western Blot were performed to determine the COL-1, TGF-β1 and αSMA expression levels (*n* = 3 per group). **(G,H)** PAS staining of paraffin-embedded renal sections (magnification, 400×, bar = 20 μm, *n* = 4 per group). **(I,J)** Masson’s trichrome staining of paraffin-embedded renal sections (magnification, 400×, bar = 20 μm, *n* = 4 per group). **(K,L)** FN antibody staining of paraffin-embedded kidney sections (magnification, 400×, bar = 20 μm, *n* = 4 per group). All data are mean ± SEM, *p < 0.05, **p < 0.01 and ***p < 0.001 vs. Control group; #*p <* 0.05 and ##*p <* 0.01 vs. DM group.

To assess the association between high salt intake and the development of DKD, we first established a mouse model of T2DM through HFD and streptozotocin (Supplementary Figure S2A). IPGTT at 2 weeks after streptozotocin injection indicated impaired glucose tolerance in diabetic mice (Supplementary Figure S2B,C). Simultaneously, 24 h urine volume and drinking water volume of T2DM mice were higher than control mice (Supplementary Figure S2D,E). Then the diabetic mice were fed a high-salt diet or a normal-salt diet for 20 weeks, followed by evaluation of the renal fibrosis. Schematics illustrating the experimental protocols are shown in (Figure 2A). Urinary sodium and serum sodium concentrations were measured at 4 weeks and 20 weeks after HSD to determine whether mice received an effective salt loading. We found that 24 h-UNa excretion of the mice in the HSD-fed DM + HNa and Control + HNa group was significantly increased (Figure 2B), but their concentrations of serum sodium rarely changed (Figure 2C). The expression levels of *Fn,* α*Sma, Col-1 and Col-3a1* genes in the renal tubules of DM group and Control + HNa group mice were significantly higher than those in the Control group (Figure 2D). Further, their expressions were increased in the DM + HNa group (Figure 2D). In addition, the changes in renal fibrosis markers COL-1, TGF-β1 and αSMA detected by western blot were consistent with the above results (Figures 2E,F). The PAS staining results indicated that HSD thickened the glomerular basement membrane and increased vacuolar degeneration of renal tubular epithelial cells in DM mice (Figures 2G,H). Furthermore, there were more widespread collagen deposition by Masson staining (Figures 2I,J) and more expression of FN by immunohistochemistry analysis (Figures 2K,L) in the kidneys of the DM + HNa group. These findings suggested that high-salt intake triggers renal fibrosis in T2DM.

### High-Salt Diet Affects Fatty Acid Oxidation, Synthesis, Transport and Release in the Renal Tubules of Diabetic Mice

**FIGURE 3:**
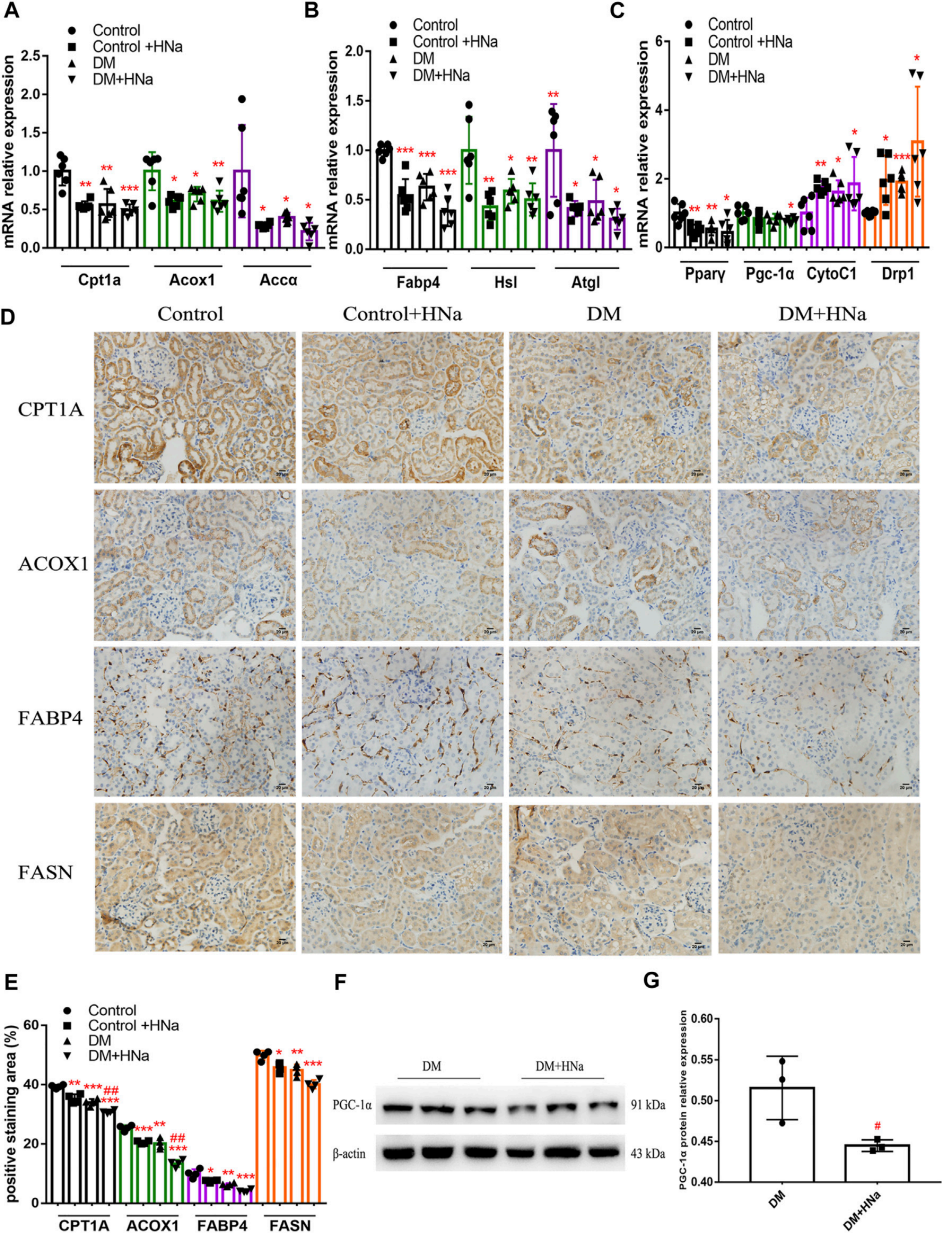
High-salt diet alters the gene and protein expression profile in renal tubules. Expression of genes involved in fatty acid metabolism **(A,B)** and mitochondrial biosynthesis **(C)** in renal tubules (*n* = 6 per group). **(D)** Paraffin-embedded renal sections were stained with CPT1A, ACOX1, FABP4 and FASN antibodies (magnification, 400×, bar = 20 μm). **(E)** Histopathological assessment of CPT1A, ACOX1, FABP4 and FASN proteins (*n* = 4 per group). PGC-1α protein expression was detected by western blot **(F)** with **(G)** relative density analysis (*n* = 3 per group). All data are mean ± SEM, *p < 0.05, **p < 0.01 and ***p < 0.001 vs. Control group; #*p <* 0.05 and ##*p <* 0.01 vs. DM group.

Fatty acid metabolism is essential to tubular epithelial cells and it is also the main source of energy in the kidney. To further investigate the mechanism underlying salt-induced exacerbation of renal interstitial fibrosis in diabetic mice, we tested the indicators of fatty acid metabolism in the renal cortex. It was indicated that HSD further restrained the fatty acid metabolism process in T2DM mice involving oxidation synthesis, transport and lipolysis as demonstrated by altered expression levels of related genes, including carnitine palmitoyltransferase 1A (*Cpt1a*), hormone sensitive lipase (*Hsl*), fatty triglyceride lipase (*Atgl*), fatty acid binding protein 4 (*Fabp4*), acetyl-CoA carboxylase α (*Accα*), and fatty acid synthase (*Fasn*) (Figures 3A,B). Even the expressions of *Pgc-1α* and *Pparγ* were down-regulated which were the important factors of mitochondrial biosynthesis (Figure 3C). Meanwhile, HSD induced increased expressions of mitochondrial biogenesis-related factors such as cytochrome C1 (*CytoC1*) and mitochondrial dynamin-related protein 1 (*Drp1*) (Figure 3C). Consistently, immunohistochemistry (Figures 3D,E) and western blot (Figures 3F,G) indicated the same changes in the protein expressions of these genes in T2DM mice fed with HSD. Additionally, the fasting blood glucose levels of HSD-fed diabetic mice were significantly reduced (Supplementary Figure S3A) and insulin sensitivity was restored (Supplementary Figures S3B,C), consistent with previous reports (Zhao et al., 2016). These data indicate that HSD induces changes in factors related to fatty acid metabolism in the renal tubules of diabetic mice.

### High Salt Suppresses Fatty Acid Metabolism Pathway to Aggravate the Damage of High-Glucose-Treated Renal Epithelial Cells

**FIGURE 4:**
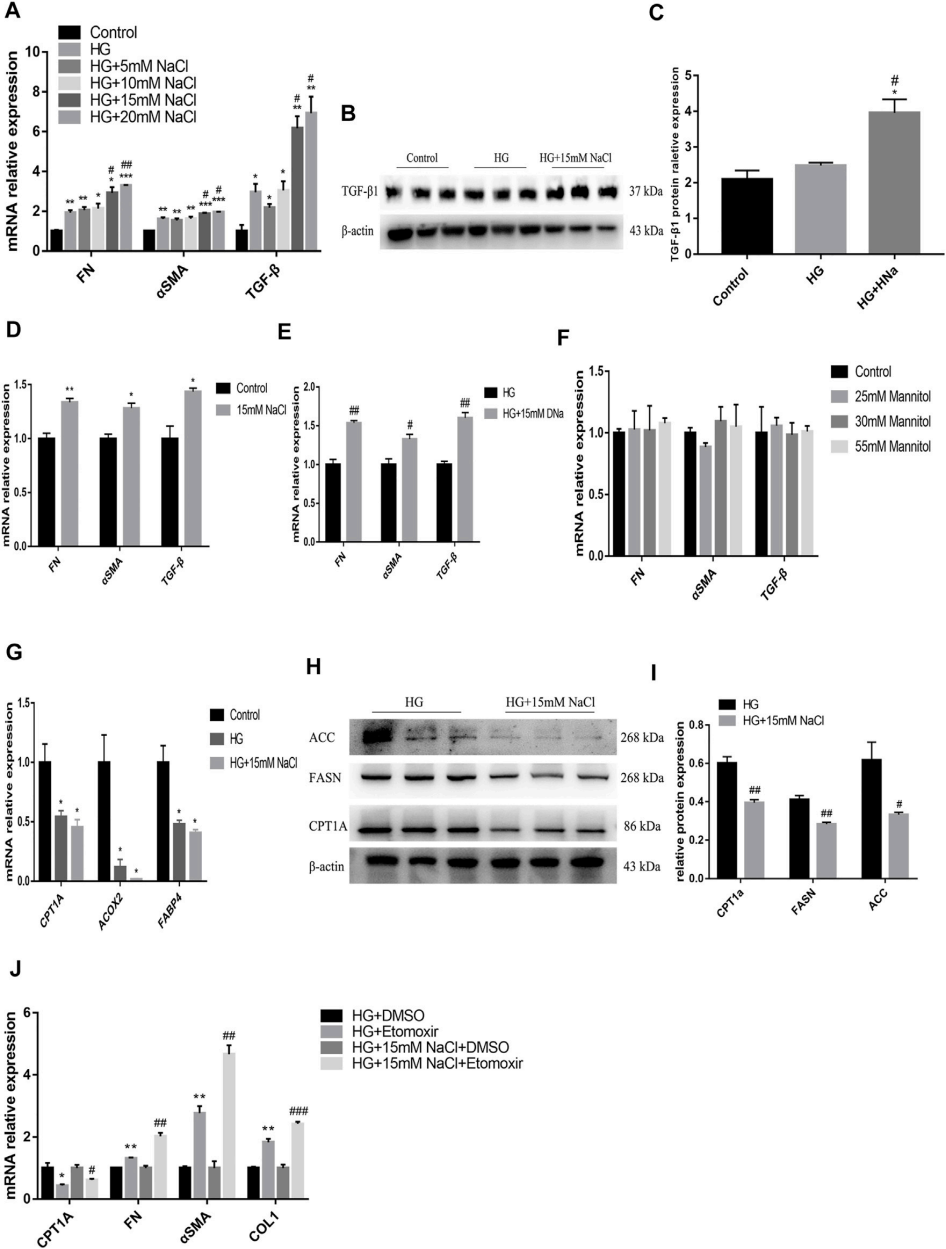
High salt suppresses fatty acid metabolism pathway to aggravate the damage of HG-treated HK-2. Expression of fibrosis-related genes **(A)** and proteins **(B,C)** in HK-2 after exposure to HG and different concentrations of NaCl. Expression of fibrosis-related genes in HK-2 respectively cultured with 15 mM NaCl **(D)**, sodium gluconate **(E)**, 25, 30 and 55 mM mannitol **(F)**. Expression of fatty acid metabolism-related genes **(G)** and proteins **(H,I)** in HK-2 with HG and 15 mM NaCl cultured. *p < 0.05, **p < 0.01 and ***p < 0.001 vs. Control group; #*p <* 0.05 and ##*p <* 0.01 vs. HG group. **(J)** Expression of CPT1A and fibrosis-related genes were detected in HK-2 treated with Etomoxir. *p < 0.05 and **p < 0.01 vs. DM + DMSO group; #*p <* 0.05, ##*p <* 0.01 and ###*p <* 0.001 vs. HG + NaCl + DMSO group. All data are mean ± SEM, *n* = 3 per group.

To detect the *in vitro* impact of salt on the renal tubules in DKD, human renal tubular epithelial cells (HK-2) were cultured for 72 h in the medium containing high glucose (HG group), high glucose and NaCl (HG + HNa group) or normal glucose and NaCl (control group), followed by examination of fibrotic indexes. The expression levels of cell fibrosis indicators *FN*, *α-SMA*, and *TGF-β* were gradually increased with the increase in NaCl concentration (Figure 4A). When the concentration of NaCl climbed to 15 mM, *TGF-β1* protein expression increased significantly by western blot (Figures 4B,C). Addition of high salt also induced cellular injury in HK-2 cultured in normal glucose (Figure 4D). The effect of high salt on HK-2 cells may be due to increased sodium, chloride, or elevated osmotic pressure. To determine the specific mechanism, we treated HK-2 with another 15 mM sodium gluconate or 25, 30 and 55 mM mannitol. The results showed that the expressions of fibrosis indicators were induced by the treatment with sodium rather than chloride (Figure 4E) and mannitol (Figure 4F). This indicates that high-salt-induced HK-2 cell damage hinges on the increase in sodium concentration, and increased osmotic pressure alone cannot change fibrotic gene expressions.

To identify the potential upstream mechanism of PTEC damage caused by high glucose and salt, we conducted an *in vitro* study in HK-2 cells, and cultured cells in a medium with 25 mM glucose and 15 mM NaCl for 72 h. Treatment with high salt further decreased the expressions of *CPT1A*, acyl coenzyme A oxidase 2 (*ACOX2*), and *FABP4* genes (Figure 4G) in high-glucose cultured cells, which was consistent with the results of *in vivo* experiments. The high glucose and salt also induced decreased protein expressions of CPT1A, FASN and ACC (Figures 4H,I). Then the cells were treated with Etomoxir, an inhibitor of FAO for 72 h, and we found that the expressions of *CPT1A* decreased and the cell fibrosis was significantly alleviated (Figure 4J). These data indicate that high salt may induce dysregulation of fatty acid metabolism in PTEC to impair renal function.

### High Salt Reduces Renal Tubular Fatty Acid Metabolism by Increasing Na+/K+-ATPase Expression in DKD

**FIGURE 5:**
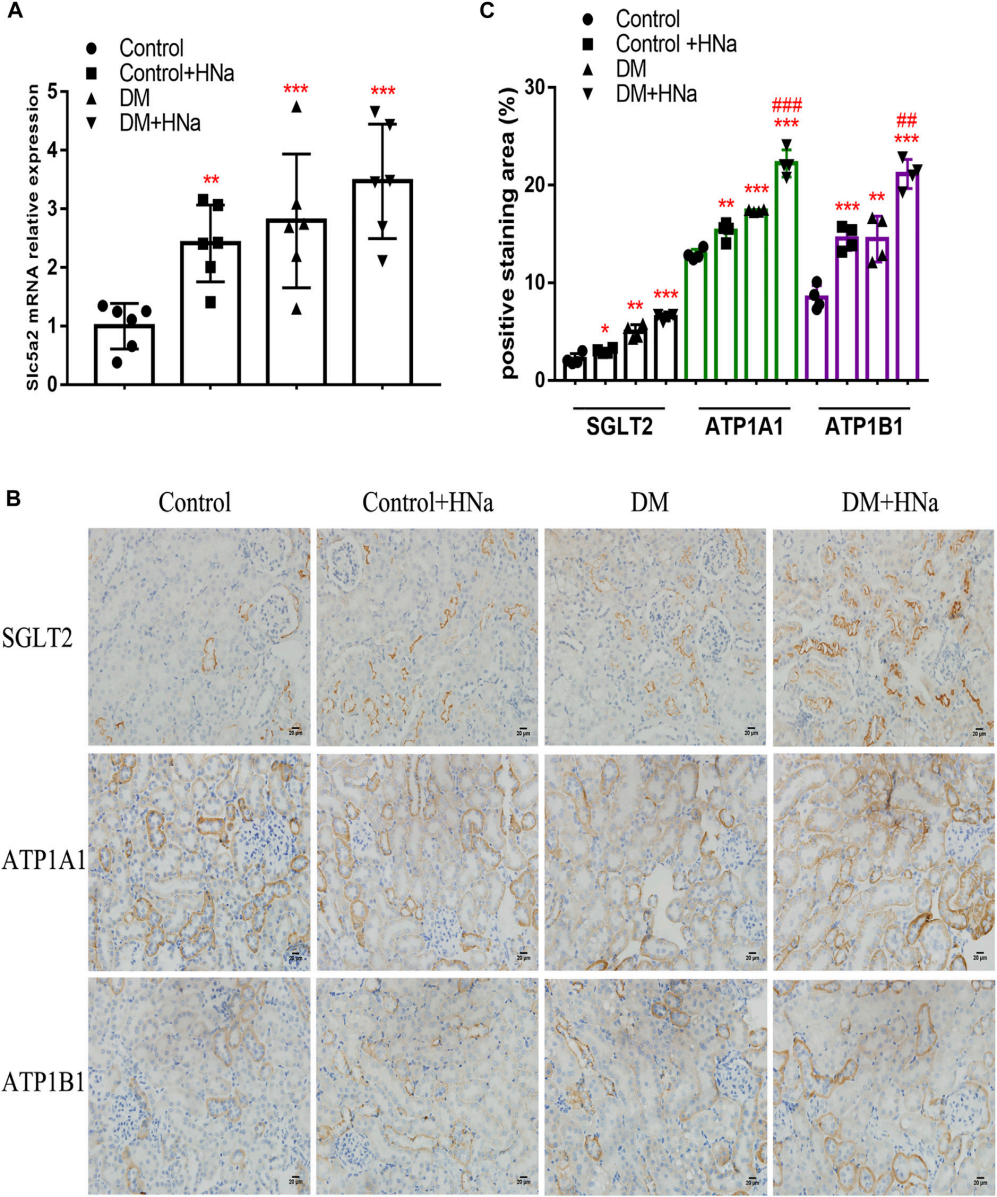
High-salt diet alters SGLT2 and Na+/K + -ATPase expression in renal tubules of DKD mice. **(A)** Expression of SGLT2 gene in renal tubules (*n* = 6 per group). **(B)** Paraffin-embedded renal sections were stained with SGLT2, ATP1A1 and ATP1B1 antibodies (magnification, 400×, bar = 20 μm). **(C)** Histopathological assessment of SGLT2, ATP1A1 and ATP1B1 proteins (*n* = 4 per group). All data are mean ± SEM, *p < 0.05, **p < 0.01 and ***p < 0.001 vs. Control group; ##*p <* 0.01 and ###*p <* 0.001 vs. DM group.

**FIGURE 6:**
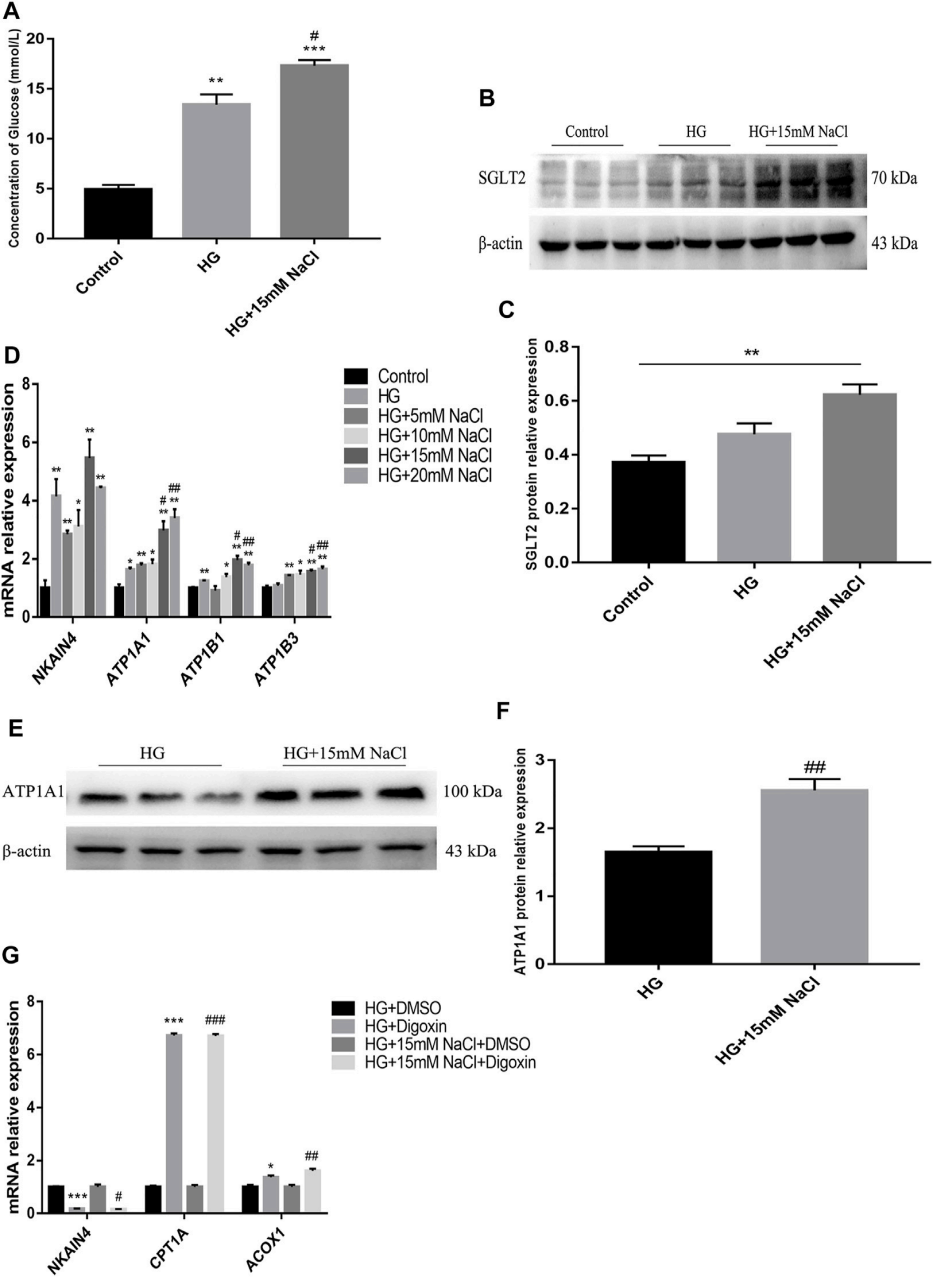
High salt alters SGLT2 and Na+/K + -ATPase expression in HG-treated HK-2. **(A)** Intracellular glucose concentration. **(B,C)** Expression of SGLT2 in HK-2 in HK-2 cultured with HG and 15 mM NaCl. **(D)** Expression of NKAIN4, ATP1A1, ATP1B1 and ATP1B3 genes in HK-2 after exposure to HG and different concentrations of NaCl. **(E,F)** Expression of ATP1A1 protein in HK-2 with HG and 15 mM NaCl cultured. *p < 0.05, **p < 0.01 and ***p < 0.001 vs. Control group; #*p <* 0.05 and ##*p <* 0.01 vs. HG group. **(G)** Expression of NKAIN4 and fatty acid metabolism related genes were detected in HK-2 treated with Digoxin. *p < 0.05 and ***p < 0.001 vs. DM + DMSO group; #*p <* 0.05, ##*p <* 0.01 and ###*p <* 0.001 vs. HG + NaCl + DMSO group. All data are mean ± SEM, *n* = 3 per group.

To clarify the potential upstream mechanism whereby high salt causes disorder of renal tubular fatty acid metabolism, we first detected increased expressions of SGLT2 (Figures 5A–C) and Na+/K+-ATPase-related indicators ATP1A1, and ATP1B1 (Figures 5B,C) in the renal cortex of HSD-fed diabetic mice. *In vitro*, the levels of intracellular glucose, SGLT2, Na+/K+-ATPase and ATP in HK-2 cells were detected. The HG + HNa group exhibited elevated glucose levels (Figure 6A) and SGLT2 protein expression in cells (Figures 6B,C). With an increase in the concentration of NaCl added to HG-treated HK-2 cells, the expression levels of the above-mentioned Na+/K+-ATPase-related indicators gradually increased (Figure 6D), which was consistent with the changes of cell fibrotic indicators (Figure 4A). ATP1A1 expression was up-regualted in the HG + NaCl group (Figures 6E,F). When the cells in HG + HNa group were administrated 5 nM Na+/K+-ATPase inhibitor Digoxin for 72 h, the expressions of Na+/K+-ATPase decreased and the expressions of Fatty acid metabolism-related genes were restored, accompanied with improvement in the cell fibrotic phenotype (Figure 6G). Collectively, these findings demonstrate suppression of Na+/K+-ATPase might alleviate the fatty acid metabolism disorder, thereby relieving renal tubular damage.

### Salt Restriction Improves the Protective Effect of Dapagliflozin on Renal Fibrosis in DKD

**FIGURE 7:**
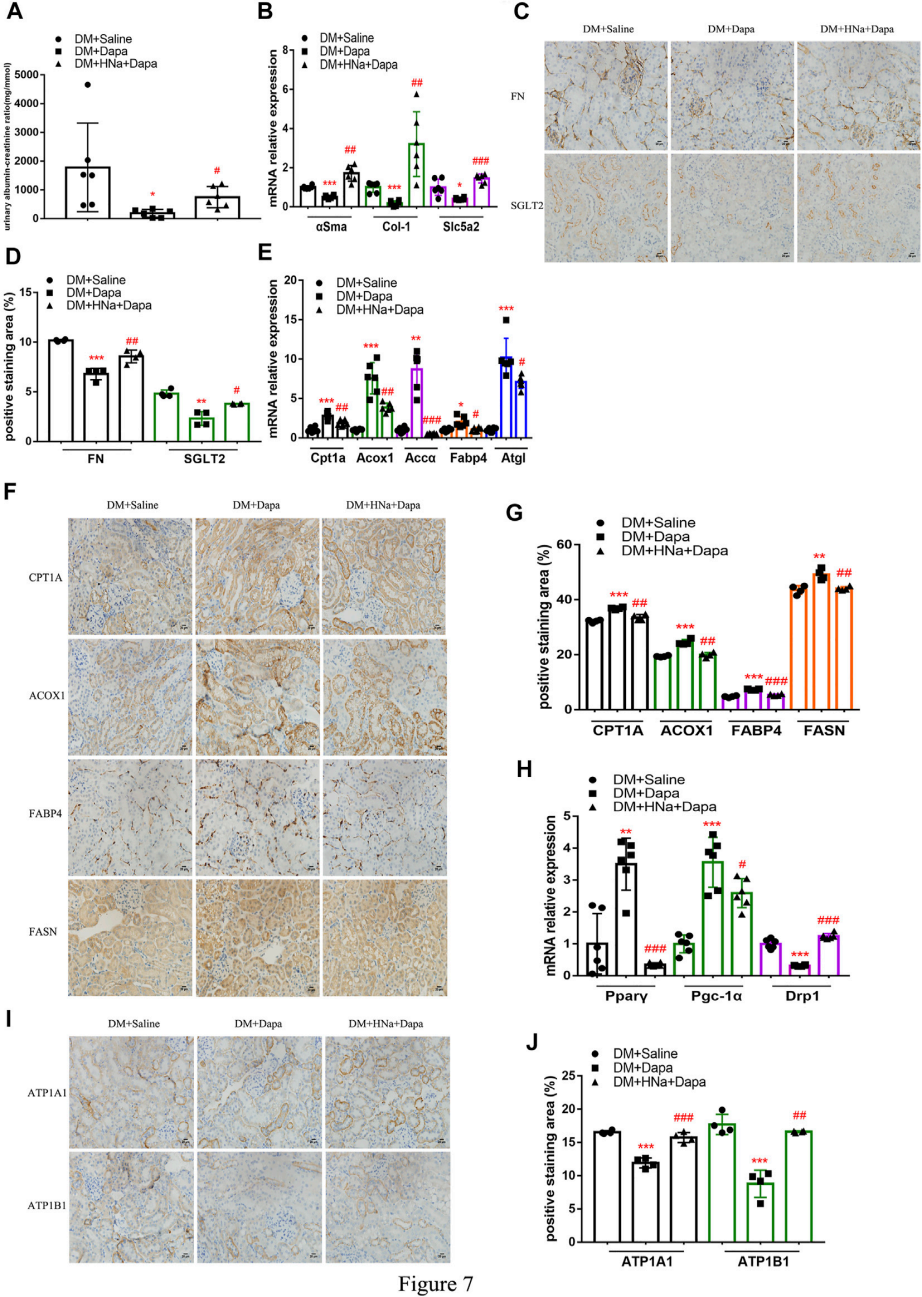
**|** High-salt diet attenuates gene and protein expression in diabetic mice treated with dapagliflozin. **(A)** The detection of ACR (*n* = 6 per group). **(B)** Expression of α*Sma, Col-1* and *Slc5a2* genes in renal tubules after dapagliflozin treatment (*n* = 6 per group). **(C)** Paraffin-embedded renal sections were stained with FN and SGLT2 antibodies (magnification, 400×, bar = 20 μm). **(D)** Histopathological assessment of FN and SGLT2 proteins (*n* = 4 per group). Expression of genes involved in fatty acid metabolism **(E)** and mitochondrial biosynthesis **(H)** in renal tubules after dapagliflozin treatment (*n* = 6 per group). **(F,I)** Paraffin-embedded renal sections were stained with CPT1A, ACOX1, FABP4, FASN, ATP1A1 and ATP1B1 antibodies (magnification, 400×, bar = 20 μm). **(G,J)** Histopathological assessment of CPT1A, ACOX1, FABP4, FASN, ATP1A1 and ATP1B1proteins (*n* = 4 per group). All data are mean ± SEM, *p < 0.05, **p < 0.01 and ***p < 0.001 vs. DM + Saline group; #*p <* 0.05, ##*p <* 0.01 and ###*p <* 0.001 vs. DM + Dapa group.

So far, several studies have noted that administration of dapagliflozin could protect kidney through improving the energy metabolism of diabetic renal tubules. There are also clinical studies suggesting that HSD affects drug’s effectiveness. ACR in diabetic mice treated with dapagliflozin was significantly reduced, which was not obvious in diabetic mice on a high-salt diet (Figure 7A). Also, the recovery of *αSma*, *Col-1* and *Slc5a2* genes in diabetic mice with HSD was weaker than that of diabetic mice (Figure 7B), as were the protein levels of FN and SGLT2 (Figures 7C,D) in diabetic mice were restored by dapagliflozin treatment; the presence of HSD decreased the effect of dapagliflozin with lower expressions. Besides, high salt decelerated the recovery of the oxidation, transport, synthesis, and release of fatty acid in the renal tubules (Figures 7E–G) and weakened the dapagliflozin’s promoting effect on restoring the levels of mitochondrial-related factors *Pparγ* and *Pgc-1α* mRNA. And the inhibitory effect of dapagliflozin on *CytoC1* (Figure 7H) and Na+/K+-ATPase-related indicators was also alleviated by HSD (Figures 7I,J).

**FIGURE 8:**
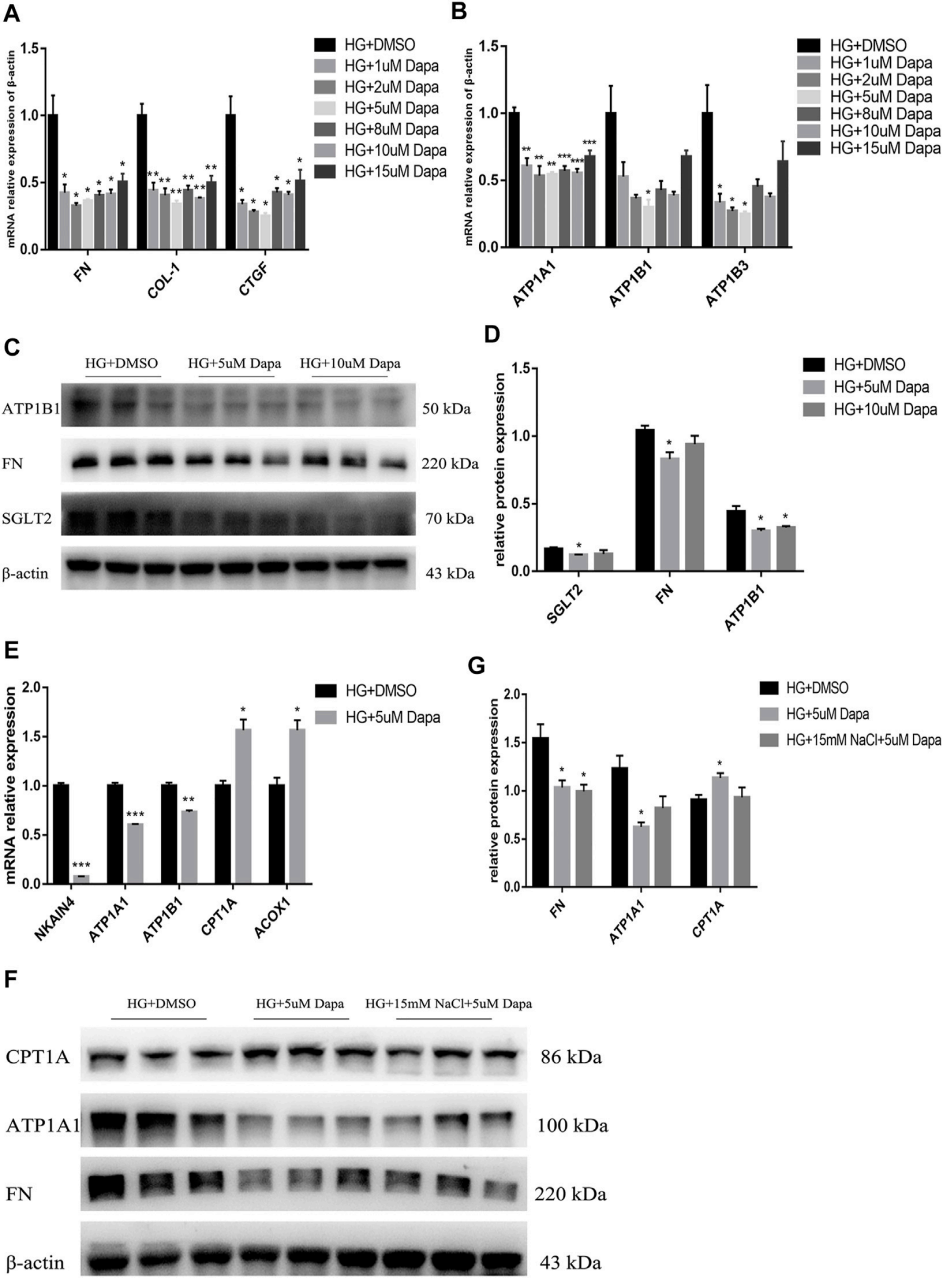
High salt attenuates gene and protein expression in HG-cultured HK-2 treated with dapagliflozin. Expression of fibrosis-related genes **(A)** and Na+/K + -ATPase-related genes **(B)** in HG-treated HK-2 after exposure to different concentrations of dapagliflozin. *n* = 4 per group. **(C,D)** Expression of SGLT2, FN and ATP1A1 proteins in HG-treated HK-2 after exposure to 5uM and 10uM dapagliflozin. *n* = 3 per group. **(E)** Expression of fatty acid metabolism-related genes. *n* = 3 per group. **(F,G)** Expression of FN, ATP1A1 and CPT1A proteins in HK-2 co-cultured with HG, 15 mM NaCl and 5 uM dapagliflozin. *n* = 3 per group. All data are mean ± SEM, *p < 0.05, **p < 0.01 and ***p < 0.001 vs. HG + DMSO group.

For investigating the direct effect of high salt on HG- and dapagliflozin-treated PTEC, HG-treated HK-2 cells were treated with 1, 2, 5, 8, 10, 15 μM dapagliflozin for 72 h. It was found that mRNA expression levels of *FN*, *COL1*, *CTGF* and *ATP1A1*, *ATP1B1* and *ATP1B3* showed inverted-U-shape dose responses to increasing dapagliflozin concentration, with 5 uM dapagliflozin exhibiting the best intervention effect (Figures 8A,B). Western blot also proved that the expressions of SGLT2, FN and ATP1B1 proteins decreased most significantly in HG- and 5 μM dapagliflozin-intervened cells (Figures 8C,D). As the expression levels of Na+/K+-ATPase decreased in dapagliflozin-treated PTEC, the expression levels of fatty acid metabolism-related genes *CPT1A* and *ACOX1* increased significantly (Figure 8E). These findings indicate that dapagliflozin reduces Na+/K+-ATPase expression in HK-2 cells and promotes fatty acid metabolism, thereby protecting the kidneys. However, the expression of ATP1A1 protein of HG + HNa group cells treated with dapagliflozin relative to HG + DMSO group was insignificantly down-regulated and there was no significant difference on CPT1A protein expression between both groups (Figures 8F,G). The above results indicate that high salt interferes with dapagliflozin to improve the energy metabolism of PTEC in DKD.

## Discussion

It is known that a healthy balanced diet is essential to prevention of diabetes, metabolic syndrome and heart disease. Herein, our study reveals three key findings about the impact of diet. First of all, we conducted a retrospective study of patients with DKD and we found that patients with macroalbuminuria have higher levels of 24 h-UNa compared with patients with microalbuminuria. Secondly, high salt intake might decrease the expressions of multiple factors of renal tubular fatty acid metabolism including oxidation, synthesis, transport, and release of fatty acid, and even cause damages on mitochondrial function. At last, our data depicted that HSD aggravates renal fibrosis in DKD mice and increases energy requirements greatly, and impairs the effects of dapagliflozin on kidneys. These findings might provide a novel mechanistic model in which high-salt diet is linked to DKD.

To determine the correlation between high salt and renal function, we established a diabetic mouse model and fed the animals with HSD. The results confirmed that the excessive salt in the diet was two to four times higher than the normal level. However, the effect of high-salt diet on the blood pressure of experimental mice in this study is unknown, which has become a limitation of this study. Previous studies have noted that high salt may predict the occurrence and development of disease including cardiovascular, DM, ESRD, and also induce TGF-β pro-fibrotic signaling independent of blood pressure (Tuomilehto et al., 2001; Hu et al., 2005; Grigorova et al., 2018). In this study, the expressions of fibrotic indicators were significantly increased in the tubules of diabetic mice. Even high salt aggravated the degree of renal fibrosis in diabetic mice. Simultaneously, the blood glucose of HSD-fed diabetic mice was elevated, which is consistent with previous reports (Zhao et al., 2016); differently, in our study HSD caused more severe renal fibrosis. This may indicate that the degree of HSD aggravating the kidney damage in diabetic mice was far greater than the risk of improving kidney damage by lowering blood glucose. Therefore, salt restriction may improve the outcome of kidney damage in DKD. Up to now, the mechanism underlying long-term HSD leading to kidney damage in DKD remains elusive. Currently, the mechanisms that lead to renal fibrosis include inflammation (Zhao et al., 2016), epithelial-mesenchymal transition (EMT) (Zhao et al., 2016), oxidative stress (Inagi et al., 2014), etc. Derkach et al., has demonstrated that abnormal salt intake affects blood metabolites, such as fatty acid, and γ-glutamyl amino acids (Derkach et al., 2017). This may reveal that high salt changes the energy utilization of kidney by inducing abnormal metabolism, thereby exacerbating kidney damage in DKD.

Impaired metabolism of fatty acids has been found in PTEC of DKD (Girard et al., 1985; Yang et al., 2018), suggesting that abnormal lipid metabolism may be involved in renal fibrosis. The results of our experiments *in vivo* revealed that in diabetic mice, high salt caused varying degrees of damage to the availability and oxidation of renal tubular fatty acids. *In vitro* experiments also have noted significant changes in genes that regulate fatty acid synthesis, release and oxidation, providing an insight into the molecular basis of changes in renal tubular metabolism. Down-regulation of *Fasn* and *Acc*α may hinder the synthesis of fatty acids, and down-regulation of *Hsl* may restrain lipolysis and mobilization during exercise, which aggravates the kidney damage. Decreased expression of Cpt1a and Acox1 prevents the mitochondrial oxidation of fatty acids, which further inhibits the availability of substrates and hinders fatty acid metabolism, and leads to renal fibrosis. In addition, down-regulation of *Fabp4* may further impair the absorption of fatty acids by the renal cortex and suppress the intracellular transport of fatty acids from the cytoplasm to the mitochondria, thus reducing cellular energy production. The impaired energetics of proximal tubules seems to be the underlying basis of lethal and sublethal tubular epithelial cell injury (Basile et al., 2012; Stadler et al., 2015). However, other studies have also pointed out that dietary sodium restriction can increase *Cpt1* expression and the content of free fatty acid (Pinto et al., 2021).

The kidney is a highly metabolically active organ with high amounts of mitochondria (Bhargava and Schnellmann, 2017). Mitochondria is mainly responsible for producing ATP for cell metabolism, which is necessary for the recovery of renal function (Bhargava and Schnellmann, 2017). Previous researches have shown that mitochondrial biogenesis and its attendant processes enhance FAO (Weinberg, 2011). Since PTEC is the most energy-consuming cell in the body, FAO and mitochondrial biosynthesis contribute to the function of the proximal tubule (Simon and Hertig, 2015). Thus, combined mitochondrial and FA metabolites showed better diagnosis values for DKD (Li et al., 2017). In the present study, the results demonstrated down-regulation of the protective factors *Pparγ* and *Pgc-1α* in the renal tubules of diabetic mice and up-regulation of the mitochondrial destructive factors *CytoC1* and *Drp1*, suggesting that mitochondrial dysfunction may contribute to the development of DKD. Besides, high intake of salt further exacerbates the imbalance between these factors in diabetic mice, leading to aggravation of kidney damage. Previous studies also suggested that high-salt aggravated renal mitochondrial dysfunctions (Wang et al., 2017). These data highlight that we may prevent mitochondrial dysfunction by reducing dietary salt intake, thereby restoring the balance of renal tubular fatty acid metabolism and improving renal function.

So far, the exact upstream mechanism underlying abnormalities of renal tubular fatty acid metabolism caused by high salt is still unclear. In fact, the vast majority of kidney energy consumption is used for sodium recovery through the basal Na+/K+-ATPase (Wang et al., 2017). The renal tubular Na+/K+-ATPase is known to impact the active transport of Na+ in the cell membrane (Katz, 1982; Jorgensen, 1986), and its expression is related to metabolic diseases (Sun et al., 2020). Our *in vivo* experiments demonstrated that HSD further increased the expression levels of SGLT2 protein and Na+/K+-ATPase in the renal tubules of diabetic mice. The results of *in vitro* experiments also confirmed increased glucose level and SGLT2 protein expression in HG + HNa group compared with that of HG group, while reabsorption of glucose requires Na+/K+-ATPase to consume ATP to pump Na+ out of the cell. Compared to control group, HG group exhibited increased expression of Na+/K+ -ATPase, secondary to the HG + HNa group. Administration of Na+/K+-ATPase inhibitor Digoxin to HG-treated HK-2 cells decreased the content of Na+/K+ -ATPase and cell fibrosis indicators, improving fatty acid metabolism and mitochondrial function. However, the addition of high salt reduced the above effects induced by Digoxin. The above data elucidate that high salt may lead to the aggravation of the fatty acid metabolism pathway through up-regulation of the renal tubular Na+/K+ -ATPase in DKD, thereby severely damaging to cells.

In diabetes, total amount of Na+ and sodium transport-dependent oxygen consumption increases (Körner et al., 1994). The acute or chronic suppression of SGLT2 could reduce proximal tubular Na + activity and hence decrease the cortical oxygen consumption (Layton et al., 2016; Vallon and Thomson, 2017; Gilbert, 2017). Dapagliflozin is effective in the prevent of progression of kidney disease with T2DM (Mosenzon et al., 2019; Pollock et al., 2019). Evidence has revealed that dapagliflozin treatment could diminish the energy requirements of the kidney by reducing the material transport in the proximal tubules and increases fat oxidation (Daniele et al., 2016). Both *in vivo* and *in vitro* experiments in this study have shown that dapagliflozin reduced kidney energy requirements and relieved the burden of kidney energy production by down-regulation of Na+/K+-ATPase, thereby improving fatty acid metabolism and mitochondrial function. A reduction of PTEC fatty acid metabolism leads to the development of renal fibrosis, so restoring fatty acid metabolism may contribute to the treatment of CKD. However, HSD is noted to interfere with drug’s effectiveness (Susic et al., 2011; Vegter et al., 2012). Consistent with previous studies, our research results revealed that dapagliflozin can significantly reduce ACR to improve renal function in diabetic mice. However, its therapeutic effect was weakened in HSD-fed diabetic mice. This is possible due to the fact that HSD leads to further renal impairment of diabetic mice. A higher dose of dapagliflozin may be able to exhibit the same effect in DKD mice as that in the treatment of diabetic mice. Therefore, we could continue to conduct more in-depth research in the follow-up experiments. *In vitro,* dapagliflozin reduced the expression of Na+/K+-ATPase, improved fatty acid metabolism, and restored the function of mitochondria in the high-glucose and high-sodium environment, but the fibrotic indicator FN was not attenuated. The above results suggest that excessive salt intake does weaken the protective effect of dapagliflozin on the renal tubules in DKD.

In conclusion, HSD increases the expression of Na+/K+-ATPase in the renal tubules of DKD mice and triggers renal tubular metabolism disorders, which hardly meet the increased cellular energy requirements and thereby may increase kidney damage. However, although dapagliflozin improves renal tubular metabolism disorders in DKD to a certain extent, high salt weakens the protective effect of dapagliflozin. Therefore, to better understand the pathogenesis and development of DKD, apart from carrying out in-depth studies on the effects of key metabolic regulators on kidney disease, we also need to attach importance to the effect of dietary salt intake on metabolic changes, pathological progress and efficacy of SGLT2 inhibitors in DKD.

## Data Availability

The original contributions presented in the study are included in the article/Supplementary Material, further inquiries can be directed to the corresponding author.

## Glossary

24h-UNa: 24h urinary sodium
ACCα: acetyl-CoA carboxylase α
ACEi: angiotensin-converting enzyme inhibitor
ACOX2: acyl coenzyme A oxidase 2
ACR: albumin-to-creatinine ratio
AcSDKP: N-acetyl-seryl-aspartyl-lysyl-proline
ARB: angiotensin II receptor blocker
ATGL: fatty triglyceride lipase
CKD: chronic kidney disease
CPT1A: carnitine palmitoyltransferase 1A
CYTOC1: cytochrome C1
DKD: diabetic kidney disease
DMEM: dulbecco’s modified eagle medium
DPP-4: dipeptidyl peptidase 4
DRP1: dynamin-related protein 1
EMT: epithelial-mesenchymal transition.
FABP4: fatty acid binding protein 4
FAO: fatty acid oxidation
FBS: fetal bovine serum
FASN: fatty acid synthase
GFR: glomerular filtration rate
GLP-1: glucagon-like peptide 1
HG: high glucose
HNa: high NaCl
HSD: high-salt diet
HSL: hormone sensitive lipase
IPGTT: intraperitoneal glucose tolerance test
IPITT: intraperitoneal insulin tolerance test
Na+/K+-ATPase: Na+/K+-adenosine triphosphatase
One-Way ANOVA: one-way analysis of variance
PAS: periodic acid-Schiff
PGC-1α: PPARγ co-activator 1α
PPARγ: peroxisome proliferator-activated receptor γ
PTEC: proximal tubular epithelial cells
RT-qPCR: real-time quantitative polymerase chain reaction
SGLT2: sodium-glucose cotransporter 2
SIRT3: sirtuin 3
T1DM: type 1 diabetes mellitus
T2DM: type 2 diabetes mellitus
