# Supplementary material for: High-Salt Attenuates the Efficacy of Dapagliflozin in Tubular Protection by Impairing Fatty Acid Metabolism in Diabetic Kidney Disease
Source: Front Pharmacol. 2021 Dec 20;12:741087. doi: 10.3389/fphar.2021.741087 (PMC8720966; doi:10.3389/fphar.2021.741087)
Supplement: Supplementary file 1 [file DataSheet1.docx]

**Supplemental tables, figures and legends**

Table S1A: The primer sequences are presented.

| Gene | Forward | Reverse |
| --- | --- | --- |
| m*-Fn* | CGAGGTGACAGAGACCACAA | CTGGAGTCAAGCCAGACACA |
| m*-αSma* | GAGGCACCACTGAACCCTAA | CATCTCCAGAGTCCAGCACA |
| m*-Col-1* | GCTCTTTTTAGATACTGTGGTGAGGAA | GTTTCCACGTCTCACCATTG |
| m*-Col-3a1* | ACAGCTGGTGAACCTGGAAG | ACCAGGAGATCCATCTCGAC |
| m*-Cpt1a* | GGTCTTCTCGGGTCGAAAGC | TCCTCCCACCAGTCACTCAC |
| m*-Cpt2* | CAATGAGGAAACCCTGAGGA | GATCCTTCATCGGGAAGTCA |
| m*-Acox1* | CTTGGATGGTAGTCCGGAGA | TGGCTTCGAGTGAGGAAGTT |
| m*-Fabp4* | GGGGCCAGGCTTCTATTCC | GGAGCTGGGTTAGGTATGGG |
| m*-Accα* | GGCCAGTGCTATGCTGAGAT | AGGGTCAAGTGCTGCTCCA |
| m*-Atgl* | AACGCCACTCACATCTACGG | GCCTCCTTGGACACCTCAATA |
| m*-Hsl* | GGAGTCTATGCGCAGGAGTG | GCTTCTTCAAGGTATCTGTGCC |
| m*-Pgc-1α* | AGTCCCATACACAACCGCAG | CCCTTGGGGTCATTTGGTGA |
| m*-Pparγ* | ATTCTGGCCCACCAACTTCGG | TGGAAGCCTGATGCTTTATCCCCA |
| m*-Drp1* | AACAGGCAACTGGAGAGGAA | GCAACTGGAACTGGCACAT |
| m*-CytoC1* | GCTACCCATGGTCTCATCGT | CATCATCATTAGGGCCATCC |
| m*-Slc5a2* | GCAACATCGGCAGCGGTCAT | GCGGAGGTACTGAGGCATTGTG |
| m*-β-actin* | CGAGCGTGGCTACAGCTTCA | AGGAAGAGGATGCGGCAGTG |
| h*-FN* | CCCTGGTGTCACAGAGGCTA | TGTATATTCGGTTCCCGGTTC |
| h*-αSMA* | ATCCTCCCTTGAGAAGAGTT | ATGCTGTTGTAGGTGGTTTC |
| h*-TGF-β* | CTTTGTACAACAGCACCCGC | CGGGTGACTTCTTTGGCGTA |
| h*-CTGF* | TATATTATATGCTGATAAGGGC | GCCAATTCGTGATTGCGAT |
| h*-COL-1* | CGATGGATTCCAGTTCGAGTATG | TGTTCTTGCAGTGGTAGGTGATG |
| h*-CPT1A* | CAGGAGTCAGTTTGGCGGTT | TCGTGGACAGGACATTGTGG |
| h*-ACOX1* | CTTGCTTCACCAGGCAACTG | CTGTCTGGGCATAAGTGCCA |
| h*-ACOX2* | ACTGAAGCCACCTATGACGC | AGTCTCCAGGCCACCATTTG |
| h*-NKAIN4* | CCACAGACAAAGCCCAGAA | TGAGGAGGTGCCAGCAGT |
| h*-ATP1A1* | ATGGAACAGACTTGAGCCG | ATTCAGGAGTAGTGGGAGGG |
| h*-ATP1B1* | GAGGGCAGCTGGAAGAAAT | GCCACTCGGTCCTGATATGT |
| h*-ATP1B3* | AACCCGACCACCGGAGAAAT | TGAGAGTCTGAAGCATAACCCA |
| h*-SLC5A2* | CTGGTCATTGGCGTTGGCTTGT | CCGATGTTGCTGGCGAAGAGAG |
| h*-18S* | AACCCGTTGAACCCCATT | CCATCCAATCGGTAGTAGCG |


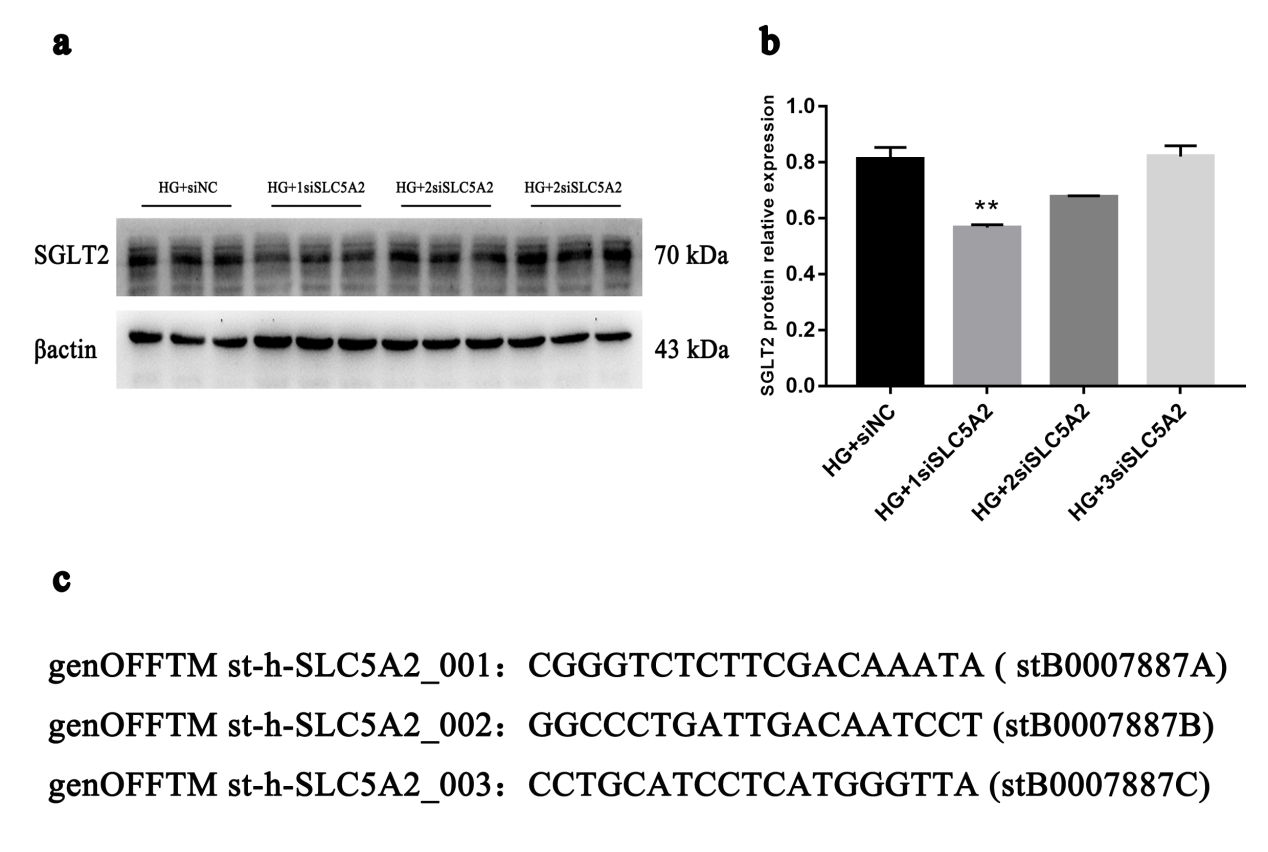


**FIGURE S1 |** Validate SGLT2 antibody. **(a,b)** Verify the three siSLC5A2 bands with the SGLT2 antibody (1:600, 24654-1-AP, Proteintech) in HK-2 cells with HG. **(c)** Silent fragments of SLC5A2. All data are mean ± SEM, ***p <* 0.01 vs. HG + siNC group, n = 3 per group.


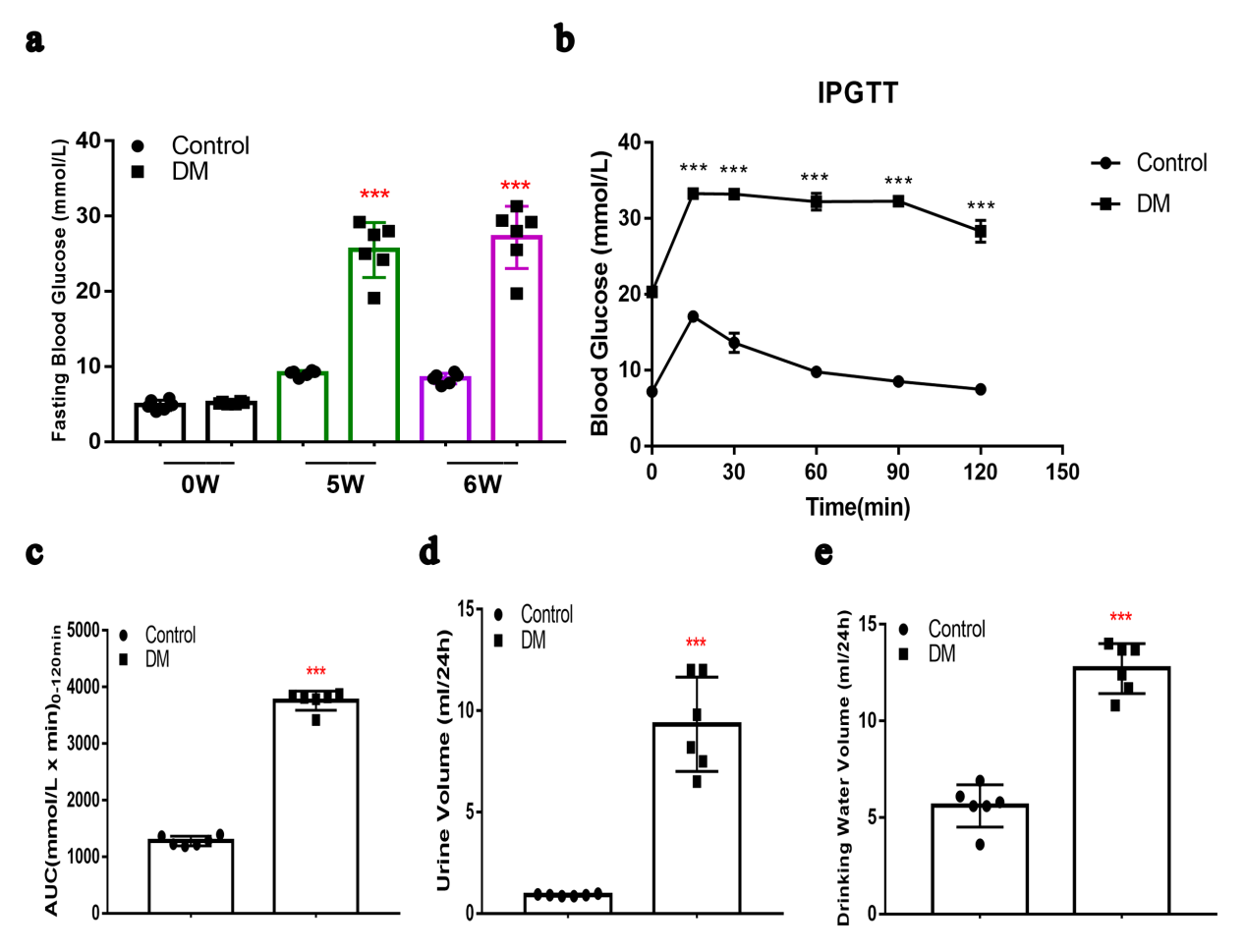


**FIGURE S2 |** Establishment of T2DM mice model. **(a)** Fasting blood glucose levels after 4 weeks HFD followed by a single injection of streptozotocin. **(b)** At 2 week after streptozotocin injection, IPGTT (2 mg/g) was performed in two groups. **(c)** AUC for glucose between 0 and 120 min. **(d,e)** At 2 week after streptozotocin injection, 24h urine volume and drinking water volume were performed in two groups. All data are mean ± SEM, ****p* < 0.001 vs. Control group, n = 6 per group.


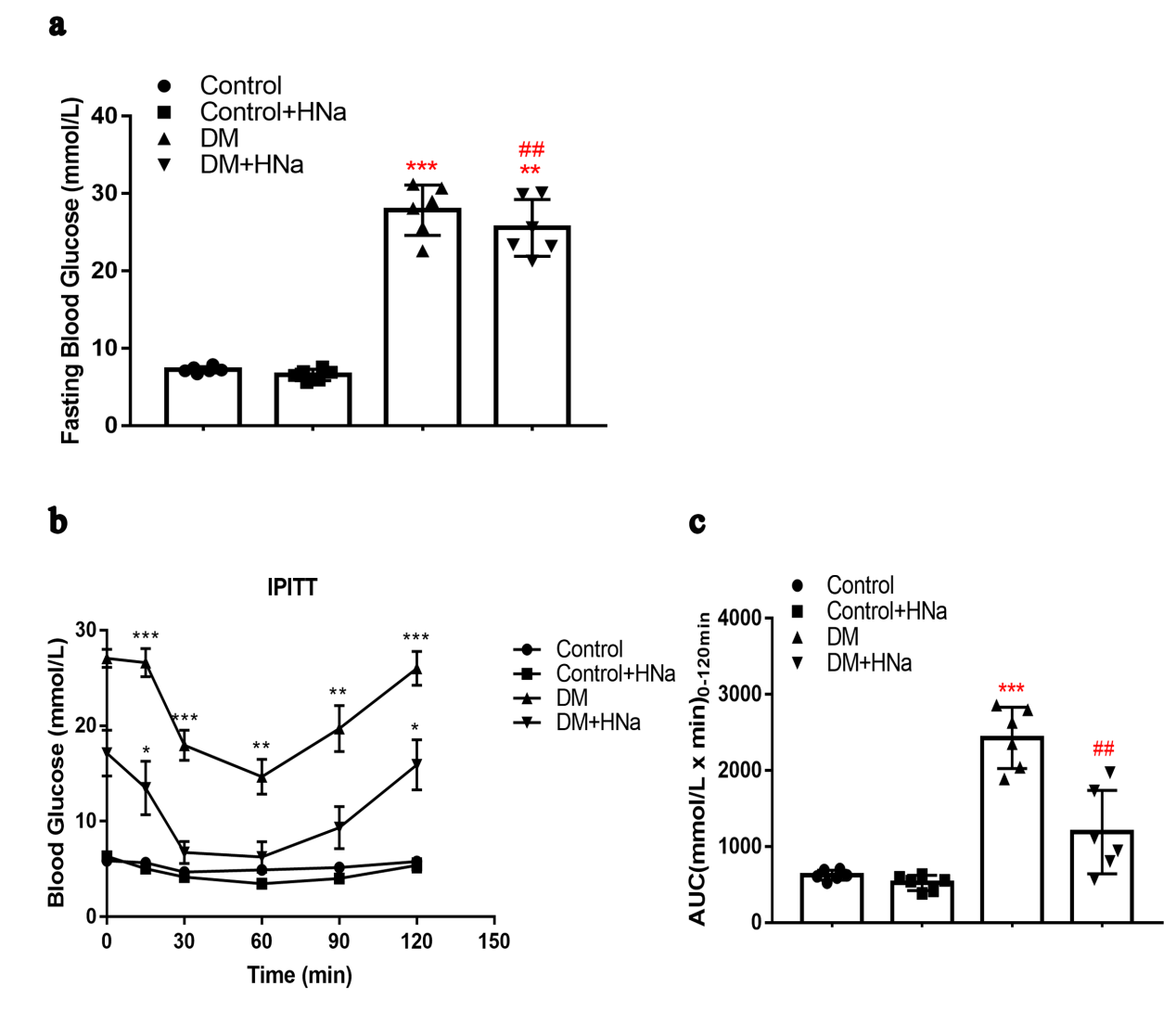


**FIGURE S3 |** Blood glucose levels of HSD-fed diabetic mice. **(a)** Fasting blood glucose levels after 20 weeks HSD. **(b)** At 20 week after HSD, IPITT (0.75 IU/kg) was performed in four groups. **(c)** AUC for glucose between 0 and 120 min. All data are mean ± SEM, **p <* 0.05, ***p <* 0.01 and ****p <* 0.001 vs. Control group; ##*p <* 0.01 vs. DM group, n = 6 per group.
